# Electrostatic Contribution of Surface Charge Residues to the Stability of a Thermophilic Protein: Benchmarking Experimental and Predicted pKa Values

**DOI:** 10.1371/journal.pone.0030296

**Published:** 2012-01-18

**Authors:** Chi-Ho Chan, Cecily C. Wilbanks, George I. Makhatadze, Kam-Bo Wong

**Affiliations:** 1 Centre for Protein Science and Crystallography, School of Life Science, The Chinese University of Hong Kong, Hong Kong, China; 2 Center for Biotechnology and Interdisciplinary Studies, Departments of Biology, Chemistry and Chemical Biology, Rensselaer Polytechnic Institute, Troy, New York, United States of America; University of South Florida College of Medicine, United States of America

## Abstract

Optimization of the surface charges is a promising strategy for increasing thermostability of proteins. Electrostatic contribution of ionizable groups to the protein stability can be estimated from the differences between the pKa values in the folded and unfolded states of a protein. Using this pKa-shift approach, we experimentally measured the electrostatic contribution of all aspartate and glutamate residues to the stability of a thermophilic ribosomal protein L30e from *Thermococcus celer*. The pKa values in the unfolded state were found to be similar to model compound pKas. The pKa values in both the folded and unfolded states obtained at 298 and 333 K were similar, suggesting that electrostatic contribution of ionizable groups to the protein stability were insensitive to temperature changes. The experimental pKa values for the L30e protein in the folded state were used as a benchmark to test the robustness of pKa prediction by various computational methods such as H++, MCCE, MEAD, pKD, PropKa, and UHBD. Although the predicted pKa values were affected by crystal contacts that may alter the side-chain conformation of surface charged residues, most computational methods performed well, with correlation coefficients between experimental and calculated pKa values ranging from 0.49 to 0.91 (p<0.01). The changes in protein stability derived from the experimental pKa-shift approach correlate well (r = 0.81) with those obtained from stability measurements of charge-to-alanine substituted variants of the L30e protein. Our results demonstrate that the knowledge of the pKa values in the folded state provides sufficient rationale for the redesign of protein surface charges leading to improved protein stability.

## Introduction

Improving protein stability is not only a matter of academic curiosity but also has potential biotechnological applications in the engineering of enzymes that are stable and active at elevated temperatures. Protein stability can be rationally improved by optimizing various types of interactions [1]–[9]. One strategy is to optimize charge-charge interactions on the protein surface [10]–[13]. Using this approach Makhatadze and co-workers have successfully increased the thermostability of several proteins [9], [14], [15] including two human enzymes, acylphosphatase and CDC42, without altering their biological activities [14].

The electrostatic contribution of an ionizable group to the Gibbs free energy of unfolding can be estimated from the differences between the pKa values in the folded and unfolded states of a protein [16]–[18]. Direct experimental measurements of the pKa value is afforded by the NMR spectroscopy, although other experimental methods such as potentiometric titration and site-directed mutagenesis (see e.g. [19]–[21]) as well as computational approaches (see e.g. [22]–[39]) can also provide estimates of the pKa values for residues of interest.

We previously demonstrated the stabilizing role of electrostatic interactions in a thermophilic ribosomal protein L30e from *Thermococcus celer* [40]–[42], and measured the changes in the Gibbs free energy of unfolding (ΔΔG_u_^mut^) for 26 charge-to-alanine substituted variants of this protein. We also calculated the energetic contribution of charge-charge interactions using different computational models, and found that these models qualitatively predict the changes in the experimental values of ΔΔG_u_^mut^ [43]. In this paper, we experimentally determined the values of pK_a_^fold^ and pK_a_^unfold^ for aspartic and glutamic acids in the *T. celer* L30e protein by NMR spectroscopy at two different temperatures (298 K and 333 K). Our results show that the pKa values and, hence, the changes in the electrostatic Gibbs free energy, ΔΔG_u_^charge^, are insensitive to temperature changes. Moreover, we showed that the experimental ΔΔG_u_^charge^ values derived from the pK_a_-shift analysis correlate strongly (r = 0.81) with ΔΔG_u_^mut^ values derived from the experimental analysis of stability of the wild type and charge-substituted variants of the L30e protein. This suggests that the knowledge of pKa values can be used as a good predictor for the effects of substitutions in ionizable residues on protein stability. Finally, our experimental pKa values were used as a benchmark to test the robustness of various computational methods of pKa calculation.

## Results and Discussion

### The 5R→K variant was created as the pseudo-wild-type for NMR experiments

Determination of the pKa values by NMR spectroscopy requires relatively high protein concentrations. In particular, it requires the protein samples to be soluble in a low ionic strength buffer. However, wild-type *T. celer* L30e crystallized readily in a low ionic strength buffer (e.g. 10 mM sodium citrate/phosphate buffer at pH 6.5) when the protein concentration was above 0.01 mM (Figure S1). The crystals formed under these conditions were used for X-ray diffraction and the structure was solved (PDB: 3N4Y) (Table S1). Structural analysis revealed that Arg-8, Arg-21, Arg-42, Arg-54, and Arg-76 are involved in crystal contacts. To reduce the crystallizability of the L30e protein, we created a quintuple variant, 5R→K, by substituting these five arginine residues with lysine. We chose these lysine substitutions because they conserve the surface charges of the L30e protein and because lysines are less likely to form crystal contacts than arginines [44], [45].

**Figure 1:**
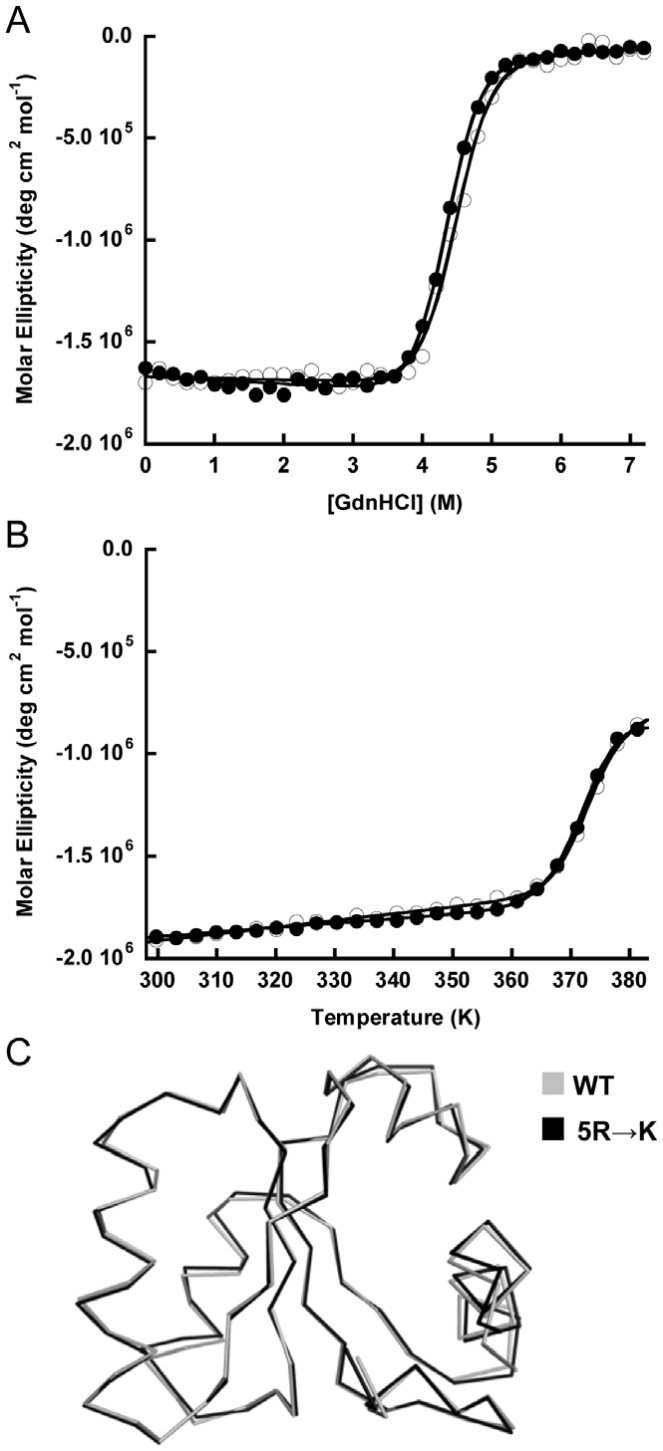
5R→K variant (L30e*) and wild-type *T. celer* L30e have similar stabilities and structures. Protein stabilities and melting temperatures of 5R→K (filled circle) and wild-type *T. celer* L30e (open circle) were determined in 10 mM citrate/phosphate buffer at pH 6.5. (A) The ΔG_u_ at 298 K of 5R→K and wild-type *T. celer* L30e were determined by guanidine-induced denaturations to be 46.4±0.3 kJ mol^−1^ and 47.5±0.3 kJ mol^−1^ respectively. (B) T_m_ of 5R→K and wild-type *T. celer* L30e were determined by thermal denaturations to be 373.2±0.1 K and 372.8±0.1 K respectively. (C) The structure of the 5R→K variant (L30e*) (black) was determined and superimposable upon that of the wild-type *T. celer* L30e (grey).

Guanidine-induced and thermal denaturation experiments showed that the 5R→K variant has essentially the same Gibbs free energy of unfolding (ΔG_u_) and melting temperature (T_m_) as the wild-type protein (Figure 1A and 1B). Moreover, the structure of the 5R→K variant (PDB: 3N4Z) (Table S1) is superimposable with the wild-type structure (Calpha r.m.s.d. = 0.47 Å) (Figure 1C). Most importantly, the 5R→K variant was very soluble (>2.6 mM) under the NMR conditions (10 mM sodium citrate/phosphate buffer), and thus we used the 5R→K variant as a pseudo-wild-type, i.e. L30e*, in subsequent NMR experiments.

### Determination of the pK_a_^fold^

**Figure 2:**
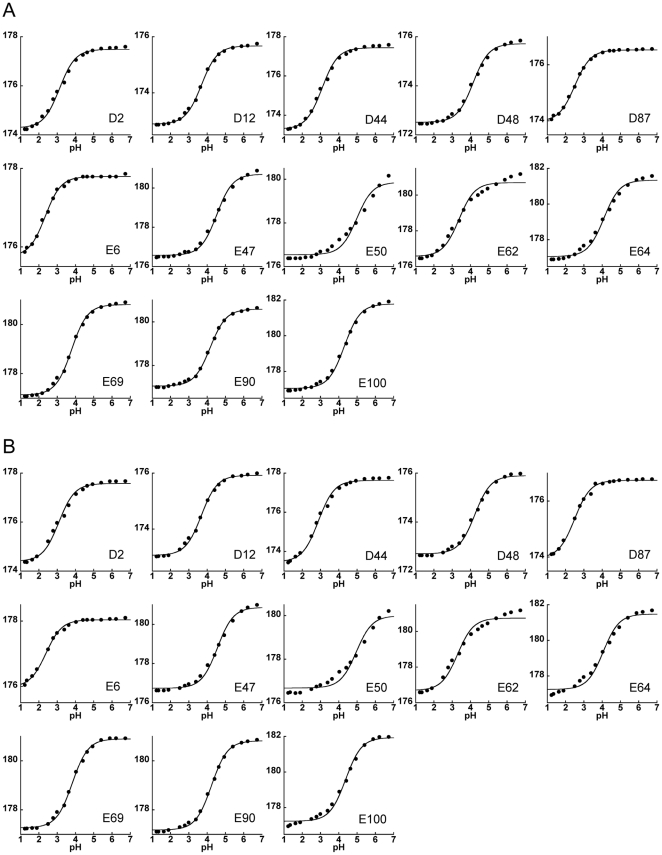
pKa values of all Asp and Glu in L30* were determined. The side-chain CO chemical shifts of Asp and Glu in native L30e* at pH 1.2–6.7 at (A) 298 K and (B) 333 K were fitted to a standard Henderson-Hasselbalch equation for determination of pKa values. The standard Henderson-Hasselbalch equation described well with all titration curves except those of E50 and E62 at both temperatures.

**Figure 3:**
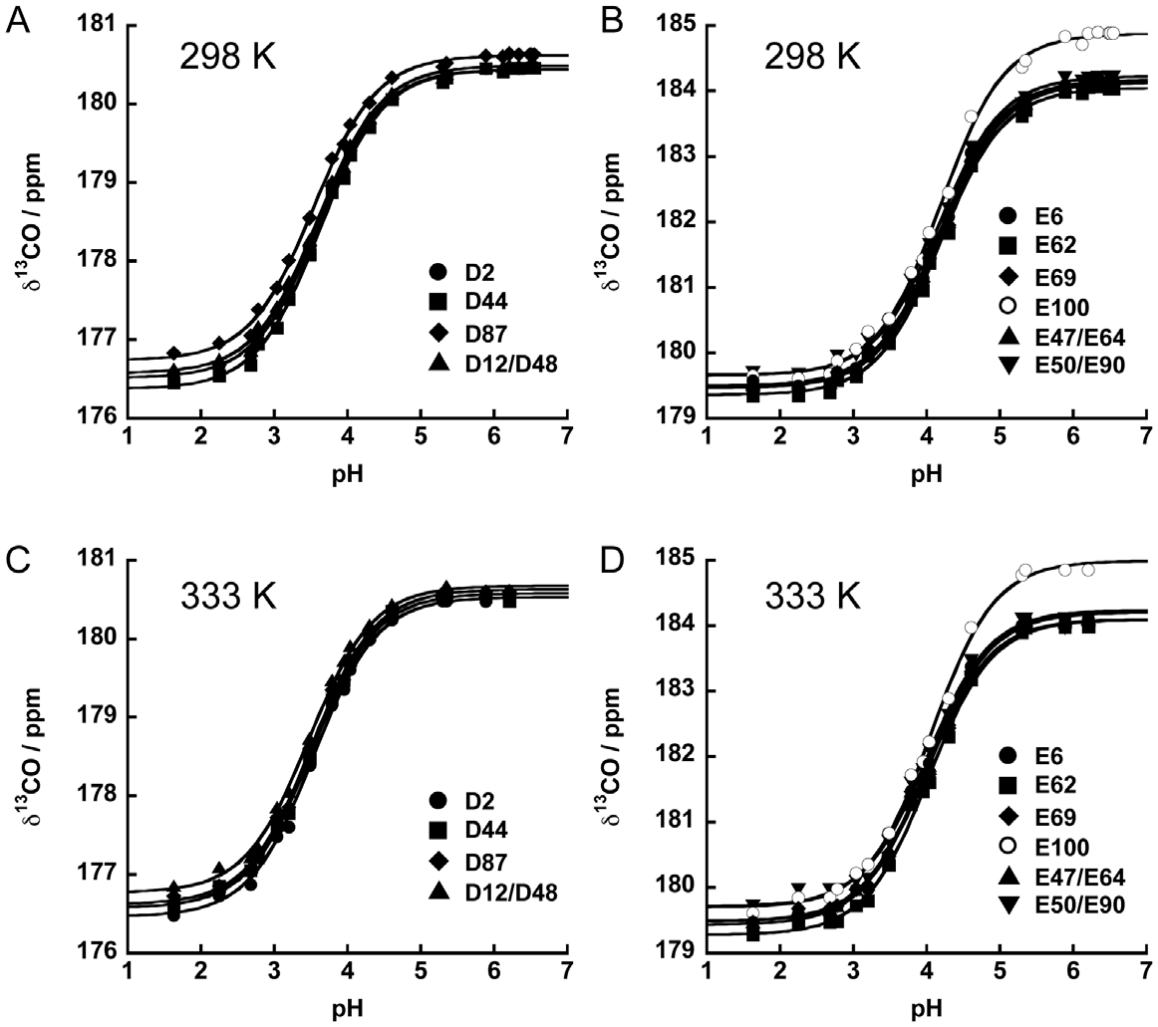
pKa values of Asp and Glu in denatured L30e* were determined. At 298 K, the side-chain CO chemical shifts of (A) Asp and (B) Glu obtained in denatured L30e* were fitted by a standard Henderson-Hasselbalch equation for determination of their pKa values. At 333 K, the pKa values of (C) Asp and (D) Glu were also obtained by the same method.

To determine the pK_a_ values of the Asp and Glu residues in the folded protein (pK_a_^fold^), the chemical shifts of the side-chain carboxyl groups (δ^13^CO) in the pH range of 1.2–6.7 were followed by a modified two-dimensional H(CA)CO experiment at two temperatures, 298 K and 333 K (Figure S2A and S2B). The changes in the chemical shifts with pH for all residues except E50 and E62 fit well to a modified Henderson-Hasselbalch equation with Hill coefficient close to 1 (Figure 2). The titration curves for E50 and E62 clearly display biphasic transitions. Interestingly, the position of the major transition of E50 approximately coincides with that of the minor transition of E62, and vice versa (). E50 and E62 are in close proximity in the crystal structure (Figure S3C), and thus these observations suggest that the ionization of E50 and E62 may be coupled [46]. To determine the pK_a_ values of E50 and E62, we used the method of global fitting of titration events (GloFTE) developed by Nielsen and co-workers [38], [47], which allows an estimate of the titration profiles for individual titratable residues (Figure 3D). The pK_a_^fold^ values of all Asp and Glu determined at 298 K and 333 K are summarized in Table 1. Comparison suggests that that the values of pK_a_^fold^ obtained at 298 K and 333 K were similar, with >0.2 unit differences observed for two residues, D44 and E64, while the rest of the pKa values remain essentially unchanged (Table 1).

**Table 1:** pK_a_ values of all Asp and Glu in native L30e* at 298 K and 333 K.

Residue	pK_a(298 K)_	pK_a(333 K)_
D2	3.13±0.04	3.09±0.05
D12	3.69±0.02	3.69±0.03
D44	3.08±0.04	2.88±0.04
D48	4.14±0.04	4.24±0.04
D87	2.50±0.02	2.49±0.03
E6	2.34±0.03	2.38±0.04
E47	4.53±0.04	4.56±0.04
E50	5.10±0.07^a^	5.00±0.06^a^
E62	3.20±0.06^a^	3.30±0.06^a^
E64	4.10±0.06	4.06±0.07
E69	3.79±0.03	3.82±0.03
E90	4.17±0.02	4.24±0.02
E100	4.31±0.03	4.38±0.05

a pK_a_ values obtained by GloFTE.

### Determination of the pK_a_^unfold^

To determine the pK_a_ values of the Asp and Glu residues in the unfolded protein (pK_a_^unfold^), the chemical shift of the side-chain carboxyl groups in the pH range of 1.2–6.7 in the presence of 5.4 M guanidine-HCl were followed by the modified two-dimensional H(CA)CO experiment (Figure S4A and S4B). The measurements were performed at two temperatures, 298 K and 333 K. The chemical shifts for D12/D48, E47/E64, and E50/E90 were degenerate. All titration curves fit well to a modified Henderson-Hasselbalch equation with Hill coefficient close to 1 (Figure 3). The summary of the obtained values of pK_a_^unfold^ is given in Table 2. The pK_a_^unfold^ values were very similar (within ±0.1 units) for each residue type: at 298 K the pK_a_^unfold^ values for Asp residues were between 3.54 and 3.62, and between 4.16 and 4.19 for Glu residues. Increasing the temperature from 298 K to 333 K resulted in a small but uniform decrease in the pK_a_^unfold^ values: 3.47–3.53 for Asp and 4.00–4.03 for Glu (Table 2).

**Table 2:** pK_a_ values of Asp and Glu in unfolded L30e* at 298 K and 333 K.

Residue	pK_a(298 K)_	pK_a(333 K)_
D2	3.62±0.01	3.53±0.02
D44	3.62±0.01	3.52±0.02
D87	3.54±0.02	3.47±0.02
D12/D48	3.56±0.02	3.46±0.02
E6	4.17±0.02	4.00±0.02
E62	4.19±0.02	4.03±0.02
E69	4.19±0.02	4.01±0.03
E100	4.19±0.02	4.06±0.02
E47/E64	4.17±0.02	4.00±0.02
E50/E90	4.16±0.02	4.02±0.03

We also determined the pKa values for model pentapeptides (pK_a_^peptide^). Model pentapeptides Ac-GG^D^/_E_GG-NH_2_ were titrated with NaOH at 298 K and 333 K in the absence and presence of 5.4 M guanidine hydrochloride. The titration curves are well described by the Henderson-Hasselbalch equation (Figure S5), and the values of pK_a_^peptide^ obtained are summarized in Table 3. The values of pK_a_^peptide^ are similar to the values of pK_a_^unfold^, suggesting that the guanidine-induced denatured state of L30e* is very close to the random coil. Importantly, the addition of guanidine hydrochloride has no significant effect on the value of pK_a_^peptide^ (Table 3).

**Table 3:** pK_a_ values of Asp and Glu in terminal protected peptides at 298 K and 333 K.

Peptide	[GdnHCl] (M)	298 K	333 K
Ac-GGDGG-NH_2_	0.0	3.69±0.03	3.61±0.02
	5.4	3.70±0.01	3.64±0.02
Ac-GGEGG-NH_2_	0.0	4.17±0.03	4.10±0.01
	5.4	4.18±0.04	4.10±0.02

### ΔpK_a_ and ΔΔG_u_^charge^ are insensitive to temperature change

**Figure 4:**
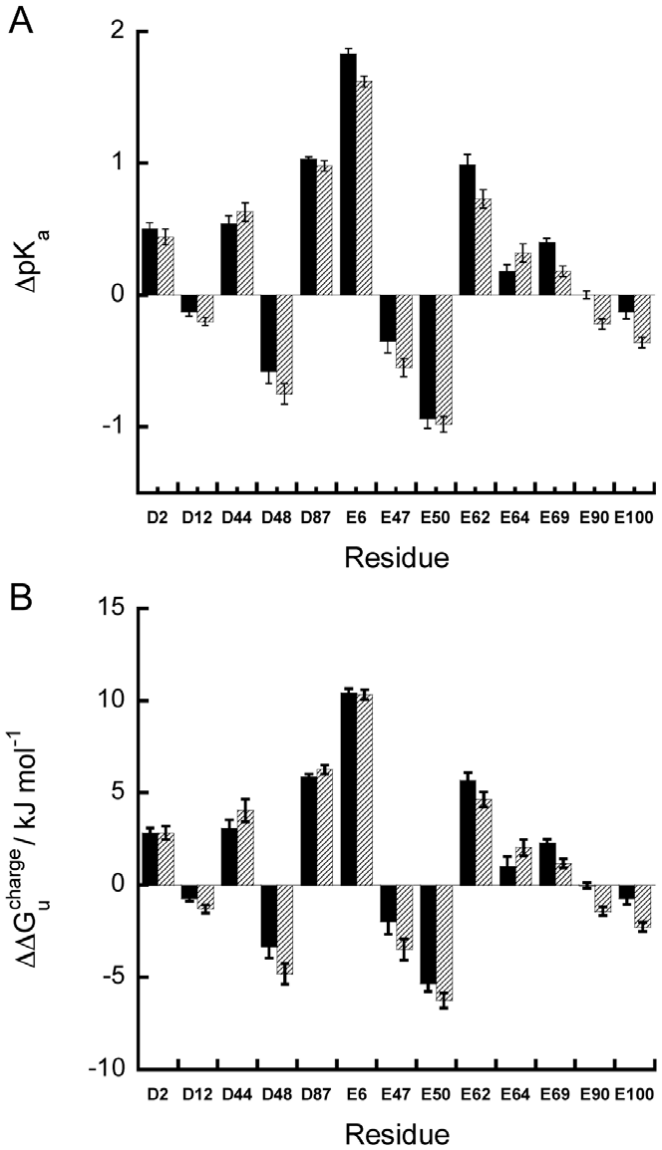
ΔpKa and ΔΔG_u_^charge^ values of Asp and Glu in L30e* are similar at 298 K and 333 K. (A) ΔpKa and (B) ΔΔG_u_^charge^ of all Asp and Glu in L30e* at 298 K (black) and 333 K (slash) were calculated. Noted the ΔpKa and ΔΔG_u_^charge^ at both temperatures are similar.

The difference in the pK_a_ values between the folded and unfolded states (ΔpK_a_ = pK_a_^unfold^−pK_a_^fold^) was used to calculate the contribution of a charged residue to the stability of the *T. celer* L30e* protein as ΔΔG_u_^charge^ = 2.303RTΔpK_a_ (Figure 4 and Table 4). The values of ΔΔG_u_^charge^ range from 10.4 kJ/mol (stabilizing) to −6.3 kJ/mol (destabilizing). In all cases, the values of ΔΔG_u_^charge^ at 298 K and 333 K were similar, suggesting that the electrostatic contribution of the charged residues to the protein stability is insensitive to temperature changes. Consistent with this observation, we have shown previously by double mutant cycle that the stabilization due to the salt-bridges E6/R92 and E62/K46 were similar at temperatures ranging from 298 to 348 K [42]. Taken together, our results suggest that favorable charge-charge interactions enhance the thermostability of proteins by up-shifting the protein stability curve.

**Table 4:** The ΔΔG_u_^ele^ values of Asp and Glu of L30e* at 298 K and 333 K.

Residue	ΔΔG_u_^ele^_(298 K)_	ΔΔG_u_^ele^_(333 K)_
D2	2.84±0.27	2.84±0.37
D12	−0.72±0.18	−1.44±0.23
D44	3.05±0.24	4.06±0.28
D48	−3.29±0.26	−4.97±0.29
D87	5.88±0.14	6.26±0.24
E6	10.43±0.23	10.31±0.26
E47	−2.05±0.26	−3.57±0.29
E50	−5.36±0.40	−6.25±0.43
E62	5.66±0.36	4.64±0.41
E64	0.40±0.36	−0.38±0.45
E69	2.29±0.19	1.17±0.25
E90	−0.02±0.15	−1.42±0.23
E100	−0.68±0.21	−2.04±0.34

### Experimental ΔΔG_u_^charge^ is highly correlated with the mutagenesis data

**Figure 5:**
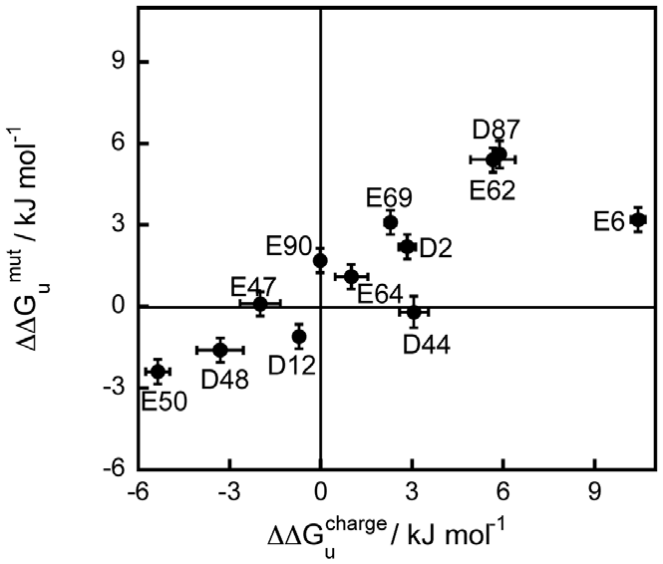
ΔΔG_u_^charge^ of Asp and Glu correlate with ΔΔG_u_^mut^. ΔΔG_u_^charge^ values obtained at 298 K from the pKa approach have a very good correlation (r = 0.81) with the ΔΔG_u_^mut^ values obtained at 298 K from charge-to-alanine mutagenesis.

The ΔΔG_u_^charge^ calculated from the changes in pKa values between the native and unfolded states at 298 K were compared with the ΔΔG_u_^mut^ obtained from stability measurements of charge-to-alanine substituted variants of the *T celer* L30e protein, ΔΔG_u_^mut^ [43] (Figure 5). It was found that the changes in ΔΔG_u_^charge^ and ΔΔG_u_^mut^ are highly correlated (r = 0.81, p-value = 0.0015). This observation suggests that ΔΔG_u_^charge^ can be used to predict the change in free energy of unfolding upon site-directed amino acid substitutions (ΔΔG_u_^mut^). Furthermore, since the pKa values in the unfolded state, pK_a_^unfold^, for each residue type (Asp and Glu) were virtually the same, the high correlation between ΔΔG_u_^charge^ and ΔΔG_u_^mut^ suggests that the knowledge of the pKa values in the native state (pK_a_^fold^) is sufficiently rational for amino acid substitutions that will modulate protein stability.

### Comparing experimental and calculated pK_a_ values

The experimental pK_a_^fold^ values were compared to those calculated by various computational models (H++, MCCE, MEAD, pKD, PropKa, UHBD) (Figure S6). These computational models were selected based on their general availability, i.e. web-based interface or free to download. They are based on different computational algorithms and utilize different models for computing pKas. Separate calculations were done using the two structural models (chain A and B) as given in the PDB entry 3N4Z. The correlation coefficients between the experimental and calculated pKa values as well as overall RMSEs and p-values are summarized in Table 5. Overall, different computational models predict pKas of the acidic residues in the *T celer* L30e* protein rather well. The p-value analysis indicates the significance of correlations with correlation coefficients ranging from 0.6 to 0.9. This suggests that for the surface residues, most current computational methods have a very good predictive power. Moreover, the correlation analysis also highlights several important aspects.

**Table 5:** Correlation coefficients between experimental and calculated pK_a_^fold^ values.

		*A*	*B*	*Aens*	*Bens*
H++	R	0.91	0.50	0.80	0.76
	RMSE	0.5	1.1	0.6	0.8
	p-value	**0.00004**	0.1	**0.002**	**0.004**
MCCE	R	0.79	0.49	0.55	0.79
	RMSE	0.8	1.0	1.0	0.7
	p-value	**0.002**	0.1	0.06	**0.002**
MEAD	R	0.89	0.81	0.89	0.82
	RMSE	0.4	0.5	0.5	0.6
	p-value	**0.0001**	**0.002**	**0.0001**	**0.001**
pKD	R	0.86	0.65	0.81	0.74
	RMSE	0.6	0.8	0.6	0.7
	p-value	**0.0003**	**0.02**	**0.0009**	**0.01**
ProPKa 2.0	R	0.85	0.58	0.70	0.72
	RMSE	0.5	0.7	0.7	0.6
	p-value	**0.0004**	**0.05**	**0.01**	**0.01**
ProPKa 3.0	R	0.88	0.61	0.65	0.73
	RMSE	0.6	0.8	0.8	0.7
	p-value	**0.0002**	**0.03**	**0.02**	**0.01**
UHBD	R	0.84	0.59	0.81	0.77
	RMSE	0.7	0.8	0.6	0.7
	p-value	**0.001**	**0.04**	**0.001**	**0.004**

The experimental pK_a_^fold^ values (298 K) were correlated with those derived from computational models. Significant correlations (p<0.05) are indicated in bold.

First, the reliability of prediction depends on the structural model used. It is apparent that in the majority of cases, correlation coefficients for the prediction based on structural model A is much better than the prediction based on the structural model B. Structure A also produces smaller RMSE between calculated and experimental values and is statistically more robust as judged by the lower p-value (Table 5). The backbone and all-atom rmsd between the structural models A and B is 0.66 and 1.24 Å, respectively, which suggests that the position of the side chains due to differences in crystal contact is leading to large differences in the computational predictions. To probe the effect of side chain flexibility on the outcome of pKa predictions, we generated an ensemble of structures starting with either of the chain A or B x-ray models (see Materials and Methods for details). Interestingly, the use of structural ensembles significantly improved correlation coefficients for the predictions based on the chain B. However, the use of structural ensembles generated based on the chain A did not improve the correlation between the experimental and calculated pKa values (Table 5). Without *a prior* knowledge of experimental pKa values, it is inconclusive to recommend whether it is a good practice to use structure ensembles or just simply use a single model in the crystal structure. Nevertheless, most prediction methods (e.g. MEAD, pKD, ProPKa, UHBD) gave significant correlation (p-value<0.05) regardless of the structural model used (Table 5).

Second, the standard errors of the prediction of pKa values using ensemble structures are relatively large in units of pH (∼0.5–0.8). However, if one thinks in the units of energy, this translates into error in ΔΔG determination of ∼2–4 kJ/mol. This highlights the difficulties in pKa predictions. One needs to be able to perform energy calculations with the accuracy better than ∼2–4 kJ/mol which is a rather difficult considering that 1 kT at 25°C is ∼2.5 kJ/mol, only twice smaller!.

Third, both physics based continuum electrostatics models and empirical models perform equally well. This is probably not surprising considering that all residues in *T celer* L30e* are located on the protein surface, and thus the contribution of solvation is largely reduced as compared to the fully buried residues. In the past, empirical models were sometimes preferred over continuum models because of the speed of calculations. However, developments in the computational algorithms and an increase in cpu power have essentially erased these differences in the speed and the use of web-based servers have made pKa calculations broadly accessible.

Fourth, there are two residues that are located in the “high charge clusters”. These are residues E50 and E62. Experimental pKa measurements show a biphasic titration profile indicating linkage in the titration properties of these residues. These effects are reasonably well predicted by the some of the computational algorithms. For example, H++ predicts strong interactions between E50 and E62, consistent with the experimental observations that there is a linkage in the titration properties of these two residues. In addition, H++ predicts the interactions of E50 with K46 and the interactions of E62 with K46/R39, which may also be responsible for abnormal titration. Using double mutant analysis we have already shown that E62/K46 pair has the energy of interactions of 3.6 kJ/mol [42]. This suggests future experiments that will measure the titration properties of the basic residues or the effect of mutations in these residues on the titration properties of E50 and E62.

### Concluding remarks

The strategy of improving the thermostability of proteins by optimizing surface charges relies on a robust method of pKa prediction and the fact that mutagenesis results can be well predicted by the pKa-shift approach. In this study, we showed that ΔΔG_u_^charge^ derived from the experimental pKa values correlates strongly with ΔΔG_u_^mut^ due to charge-to-alanine substitutions. This observation suggests that the knowledge of the pKa values (obtained either by experimental or computational methods) is sufficiently rational for the optimization of protein surface charges. Our experimental pKa values were also served as a benchmark to test the robustness of various computational methods of pKa prediction. Most current computational methods gave good prediction of pKa values. The encouraging fact is that all of these methods are generally available, either via a web-interface or free to download, making rational redesign of the protein surface charges accessible to all protein engineers.

## Materials and Methods

### Cloning, expression and purification

The DNA coding for the 5R→K variant of L30e was synthesized by Mr. Gene GmbH (http://mrgene.com) and was sub-cloned in the expression vector pET3d (Novagen). All unlabeled and 13C/15N labeled protein samples were expressed and purified as described previously [41], [43].

### Measurement of thermodynamics stability

Thermal-induced denaturation experiments were performed in 10 mM citrate/phosphate buffer at pH 6.5 using 20 µM protein concentration. The melting temperature (T_m_) of the protein was determined as described [42]. Guanidine-induced denaturation experiments at 298 K were performed in the same buffer. The Gibbs free energy of unfolding (ΔG_u_) was determined as described [40].

### Crystallization and structure determination

Crystals of wild-type T. celer L30e were grown by dialyzing 0.1 mM protein into 10 mM citrate/phosphate buffer pH 6.5 at 277 K. Crystals of the 5R→K variant were grown by the sitting-drop-vapor-diffusion method using 1.6 M Na/K phosphate buffer at pH 7.5 and 289 K. Data collection and structure determination were performed as described [42].

### Determination of pK_a_

For the folded state of the 5R→K variant, sequential assignment of backbone resonances was obtained by C^α^ and C^β^ connectivities generated by the HNCACB and CBCA(CO)NH experiments at pH 6.7 at 298 K and 333 K. The side-chain resonances were obtained from ^15^N-TOCSY-HSQC, HC(CCO)NH, (HC)C(CO)NH, and HCCH-TOCSY experiments. The side-chain carboxyl carbon of Asp/Glu (δ_exp_) at pH 6.7 at 298 K and 333 K were assigned using a modified three dimensional HCACO experiment, which correlates side-chain CO^γ/δ^ with H^β/γ^ and C^β/γ^ resonances [48]. The assignments of the δ_exp_ at other pH were obtained by tracing the peak shift in the modified two dimensional H(CA)CO experiments collected in the pH range of 1.2–6.7.

For the pKa determination in the unfolded state of the 5R→K protein, the protein was denatured in 5.4 M guanidine-HCl for all NMR experiments. Sequential assignment of backbone resonances pH 6.4 at 298 K and 333 K was obtained by the connectivities of C^α^ and C^β^ generated by HNCACB and of *d*_NN(i,i+1)_ NOEs generated by the modified HSQC-NOESY-HSQC experiment [49]. The assignments of side-chain resonances and the δ_exp_ at pH 6.4 at 298 K and 333 K were obtained as described above. The δ_exp_ in the pH range of 6.4–1.6 were also obtained by tracing the gradual peak shift associated with the pH change. The pH of all protein samples were measured by glass pH-electrode (Beckman Coulter φ510) before NMR experiments. The pH of all samples was re-measured after performing each NMR experiments, and the variation of the pH was less than 0.05 units.

pK_a_^fold^ and pK_a_^unfold^ were determined by fitting the δ_exp_ into a modified Henderson-Hasselbalch equation: δ_exp_ = [δ_A_+δ_B_10^n(pH–pKa)^]/[1+10^n(pH–pKa)^], where δ_A_ and δ_B_ are chemical shifts for the protonated and deprotonated residue respectively. The Hill coefficient, n, was set to be a free parameter during data-fitting. With the exception of the titration profiles for E50 and E62 in the folded state, the Hill coefficient in all cases was close to 1. According to F-test, a simpler model with Hill coefficient set equal to 1 was used in the data fitting.

For E50 and E62, their pK_a_^fold^ were determined by global fitting of titrational event (GloFTE) using pKaTool [38], [47]. The pH step was set to 0.1 and the Monte Carlo step number was set to 300. The titration curves were calculated based on the explicit evaluation of the Boltzmann sum. The pK_a_^fold^ was determined by finding the pH where the titratable group is half-protonated [38].

### pH titration of side-chain carboxyl groups in model peptides

Termini protected glycine-based pentapeptides Ac-GGD/EGG-NH_2_ were purchased from GL Biochem (Shanghai). 40.2 mg lyophilized peptide powder of Ac-GGDGG-NH_2_ and 41.6 mg lyophilized peptide powder Ac-GGEGG-NH_2_ were dissolved in 10 ml nano-pure water using 10 ml volumetric flask. Before pH titration, the solution was equilibrated at 298 K and 333 K for 15 minutes. The pH value of the solution was continuously measured by the calibrated pH meter using glass electrode (Beckman Coulter φ510). Commercially purchased 1.0 M NaOH (Sigma) was used for the titration. 10 µl of NaOH was added to the solution stepwise until a pH value greater than 8.0 was reached.

pK_a_^peptide^ were determined by fitting the NaOH concentration in the solution into a modified Henderson-Hasselbalch equation: C_exp_ = 1000×10^−pH^−[10^−pKa^×C_peptide_/(10^−pH^+10^−pKa^)]−C_offset_, where C_exp_ is the NaOH concentration in the solution in mM, C_peptide_ is the peptide concentration, and C_offset_ is the OH^−^ concentration in the solution before a given titration step.

### Calculation of pKas

We used seven different software packages that predict the pKas of amino acid residues. In all cases the default parameter sets were used. The coordinate files were supplied in the PDB format using individual models A and B from the X-ray structure of L30e* protein (PDB:3N4Z). To probe the effects of side-chain conformational flexibility, these two structural models were used as a starting template for generating a structural ensemble within the MODELER environment [50]. Calculations were performed on each individual structure of the ensemble, and the results were averaged.

MEAD v2.2.9 [23] (http://hospital.stjude.org/mead_filerequest/request.html) solves linearized Poisson-Boltzmann to perform various electrostatic calculations including pKas of amino acid residues in proteins.

H++ [24] (http://biophysics.cs.vt.edu/) uses the MEAD engine [23] to solve the Poisson-Boltzmann equation, but in addition uses a “smeared charge” concept that places partial charges on several atoms in the ionizable side chain instead of one single atom.

pKD [25] (http://enzyme.ucd.ie/cgi-bin/pKD/server_start.cgi) uses the Poisson-Boltzmann solver DELPHI II [26] together with the optimization of a hydrogen bonding network as implemented in the WHATIF software package [27].

PROPKA_2.0 [29] and PROPKA_3.0 [28] (http://enzyme.ucd.ie/cgi-bin/pKD/server_start.cgi) estimate the shift in the pKa arising from hydrogen bonds, relative burial and coulombic interactions. These contributions are parametrized to fit experimentally measured ΔpKa deposited into pKa databases (e.g. http://www.sci.ccny.cuny.edu/~mcce/index.php and http://www.ddg-pharmfac.net/ppd/PPD/pKahomepage.htm).

UHBD [30] (http://mccammon.ucsd.edu/uhbd.html) uses the finite difference Poisson–Boltzmann (FDPB) method to calculate electrostatic interactions. Energies of interactions were combined with the hybrid method to calculate pKas [31].

MCCE Multi Conformer Continuum Electrostatics (version 2.5) software package uses Monte-Carlo sampling of different side-chain rotamers in conjunction with the FDPB calculations using the DELPHI software package and PARSE solvation [32].

## Supporting Information

Figure S1 **Wild-type** ***T. celer*** **L30e crystallized in low ionic strength buffer (10 mM citrate/phosphate buffer, pH 6.5.**
(PDF)

Figure S2 Assignment of side-chain carboxyl carbon of Asp and Glu in native L30e*.
(PDF)

Figure S3 **Titration of Glu-50 and Glu-62 was coupled.** Two transitions were observed in the titration curves of Glu-50 and Glu-62 at (A) 298 K and (B) 333 K, of which the major transition of one residue corresponds to the minor transition of another. This observation suggests that the protonation of Glu-50 and Glu-62 are coupled, as these two residues are in close proximity to each other (C). The pKa values of Glu-50 and Glu-62 at (D) 298 K and (E) 333 K were determined by fitting their titration curves simultaneously by the method of global fitting of titration events (GloFTE).
(PDF)

Figure S4 Assignment of side-chain carboxyl carbon of Asp and Glu in unfolded L30e*.
(PDF)

Figure S5 **Determination of pKa^peptide^.** Titration curves of model peptides (A, C, E, G) Ac-GGDGG-NH_2_ and (B, D, F, H) Ac-GGEGG-NH_2_ in the presence of (A–D) 5.4 M and (E–H) 0 M guanidine HCl (A, B, E, F) 298 K and (C, D, G, H) 333 K. All of the titration data were fitted to the standard Henderson-Hasselbalch equation for determination of the pKa values of Asp and Glu in model peptides.
(PDF)

Figure S6 **Correlation of experimental and predicted pKa values.** pKa values were calculated using several different software packages indicated on each plot. Left panels for each method are based on chain A of the crystal structure of L30e* (PDB code: 3N4Z), right panels for each method are based on chain B. Blue circles represent the results of based on x-ray model (A or B). Red squares represent the results of ensemble calculations based on structures A or B. Red and blue lines are the corresponding linear correlations. Thin black line shows the perfect correlation. See Materials and Methods section for the details of the calculations.
(PDF)

Table S1 **Statistics for structure determination of wild-type T. celer L30e and 5R→K variant.**
(PDF)
