# Supplementary figures and images for: Electrostatic Contribution of Surface Charge Residues to the Stability of a Thermophilic Protein: Benchmarking Experimental and Predicted pKa Values
Source: PLoS One. 2012 Jan 18;7(1):e30296. doi: 10.1371/journal.pone.0030296 (PMC3261180; doi:10.1371/journal.pone.0030296)

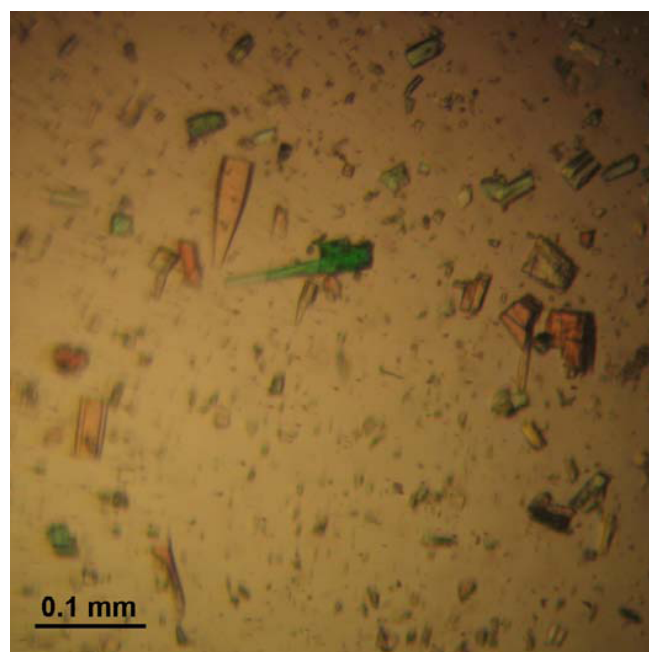

Supplement: Figure S1 — Wild-type T. celer L30e crystallized in low ionic strength buffer (10 mM citrate/phosphate buffer, pH 6.5. (PDF) [file pone.0030296.s001.pdf]

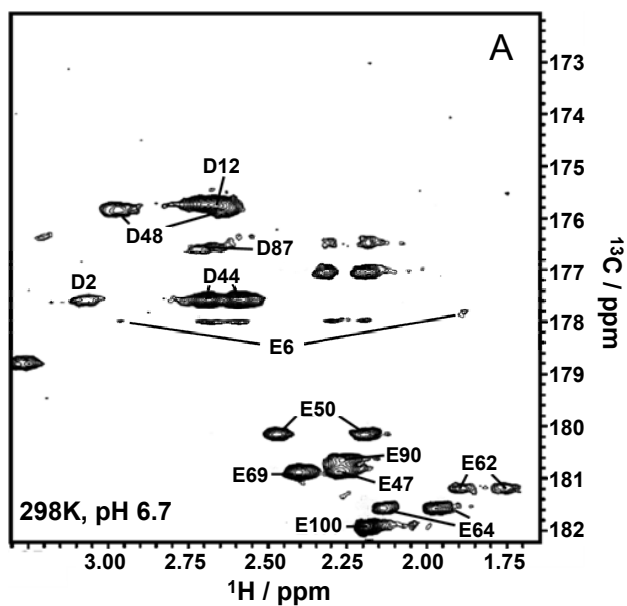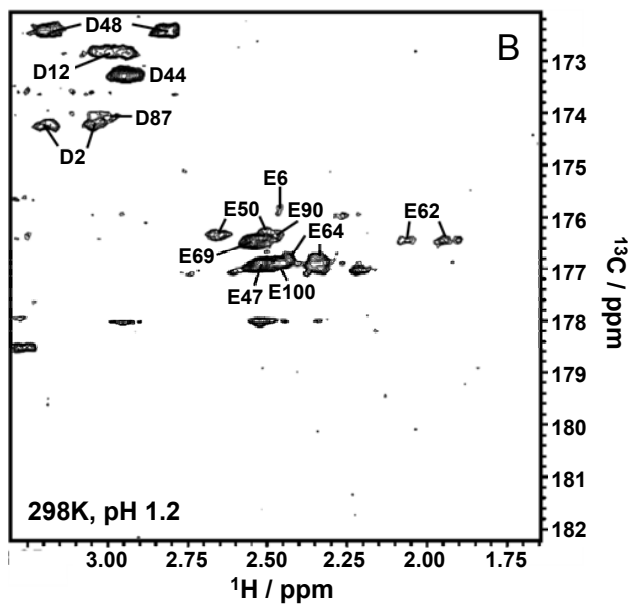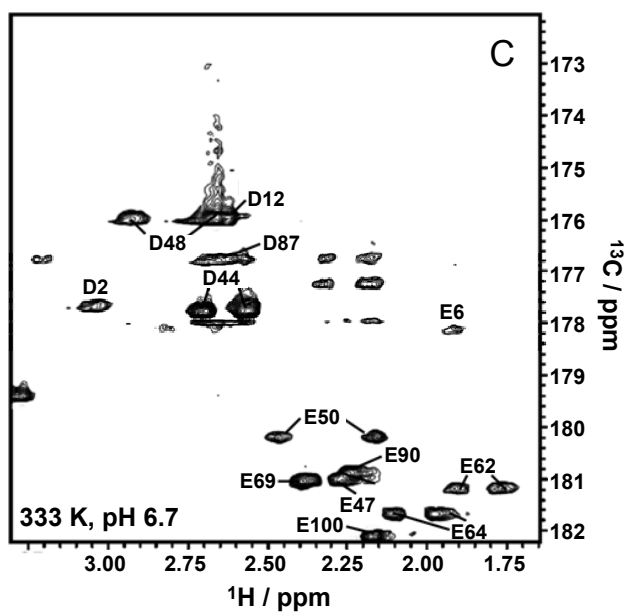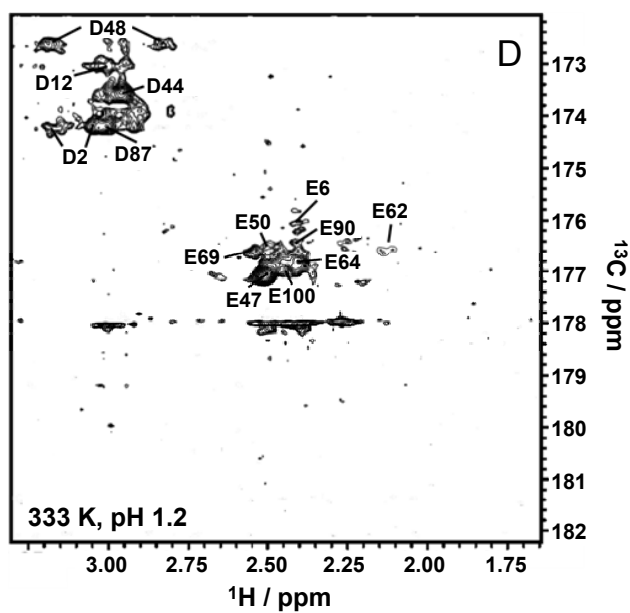

Supplement: Figure S2 — Assignment of side-chain carboxyl carbon of Asp and Glu in native L30e*. (PDF) [file pone.0030296.s002.pdf]

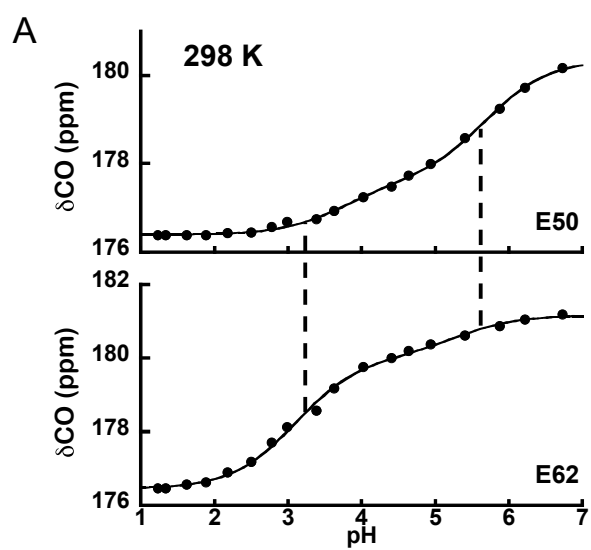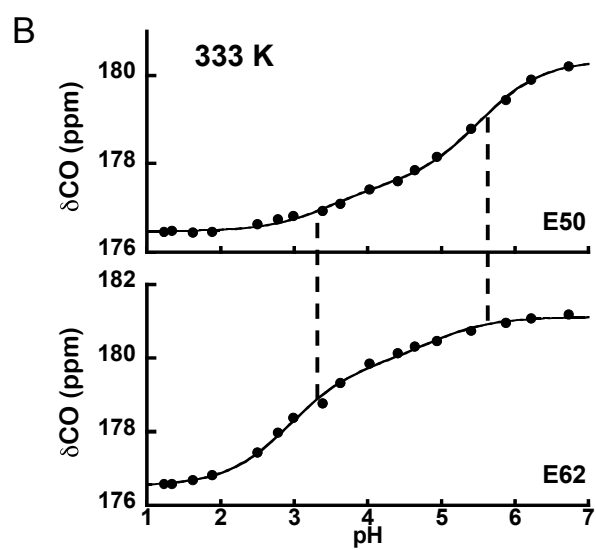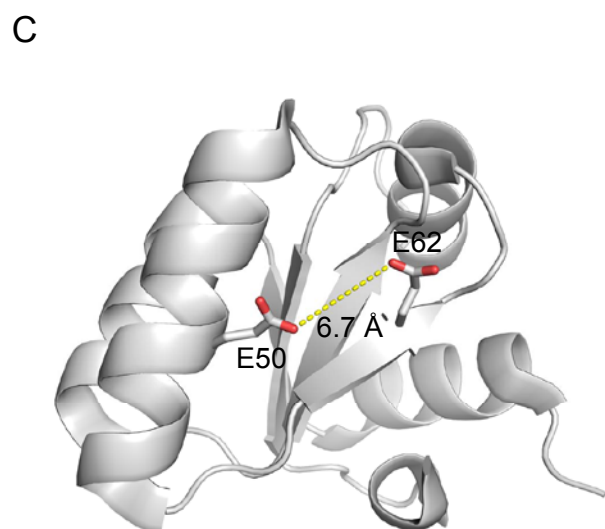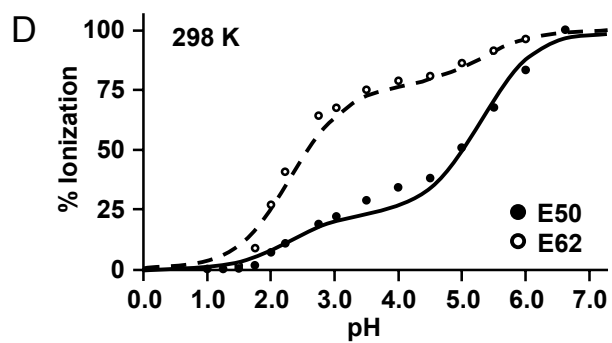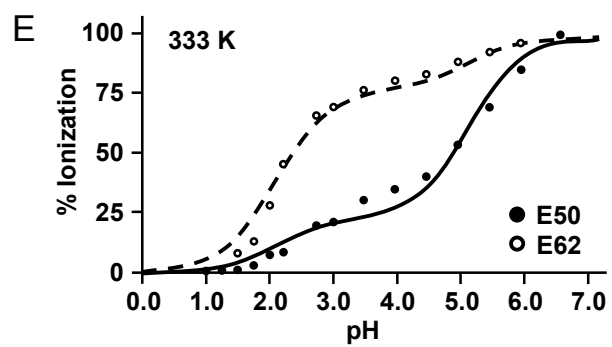

Supplement: Figure S3 — Titration of Glu-50 and Glu-62 was coupled. Two transitions were observed in the titration curves of Glu-50 and Glu-62 at (A) 298 K and (B) 333 K, of which the major transition of one residue corresponds to the minor transition of another. This observation suggests that the protonation of Glu-50 and Glu-62 are coupled, as these two residues are in close proximity to each other (C). The pKa values of Glu-50 and Glu-62 at (D) 298 K and (E) 333 K were determined by fitting their titration curves simultaneously by the method of global fitting of titration events (GloFTE). (PDF) [file pone.0030296.s003.pdf]

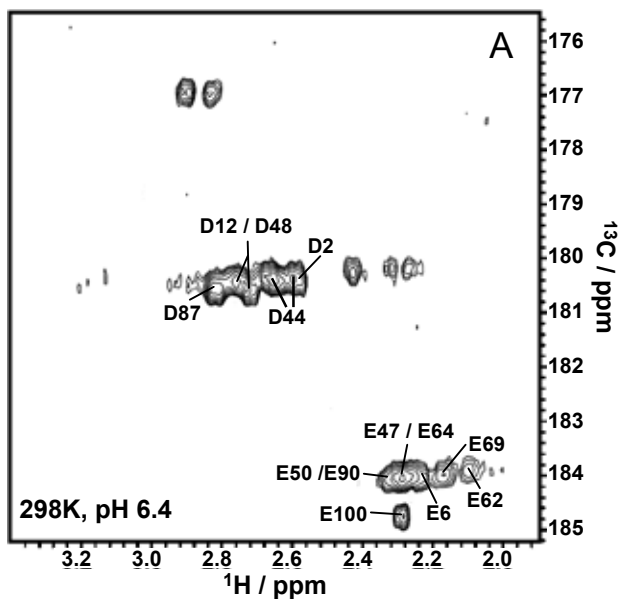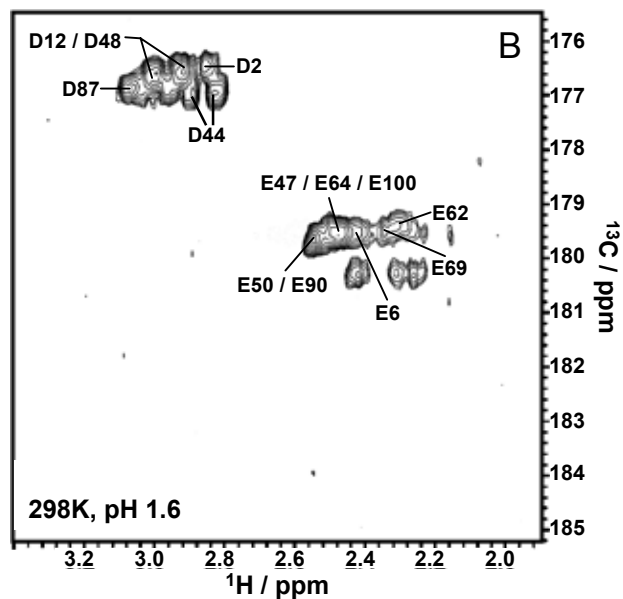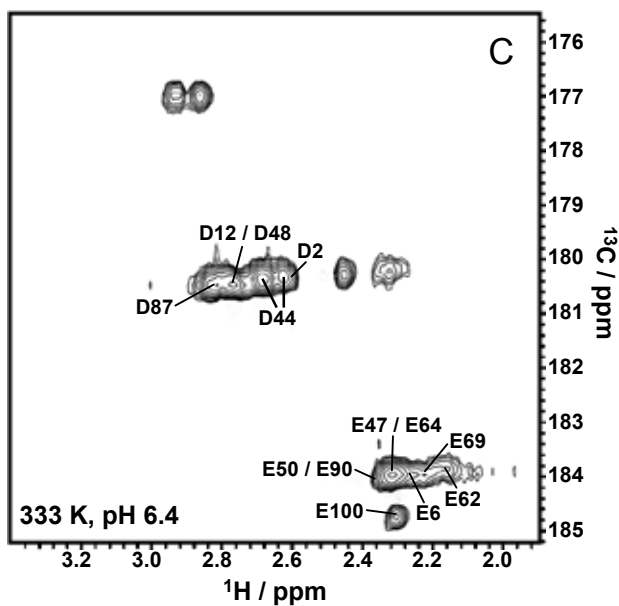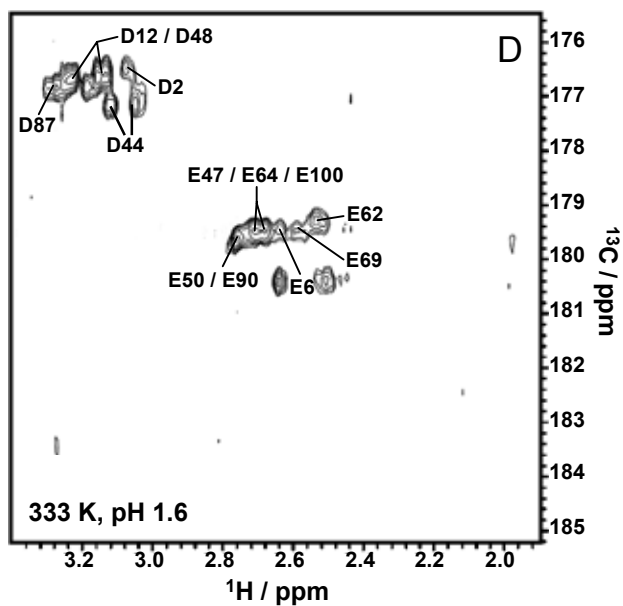

Supplement: Figure S4 — Assignment of side-chain carboxyl carbon of Asp and Glu in unfolded L30e*. (PDF) [file pone.0030296.s004.pdf]

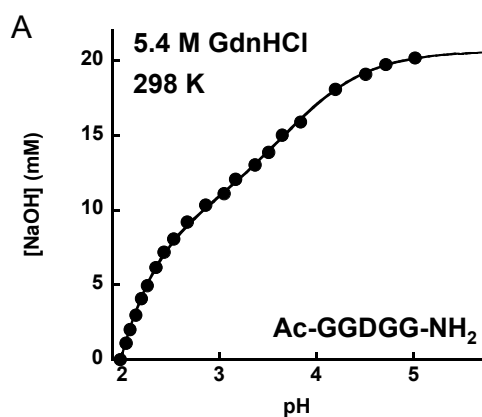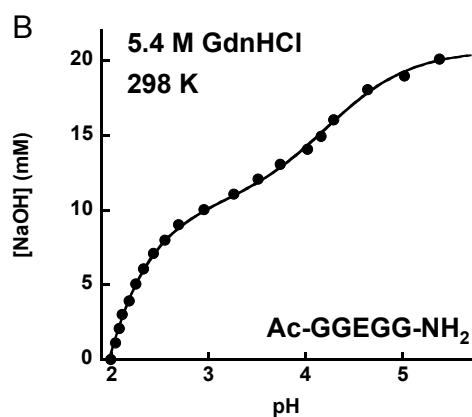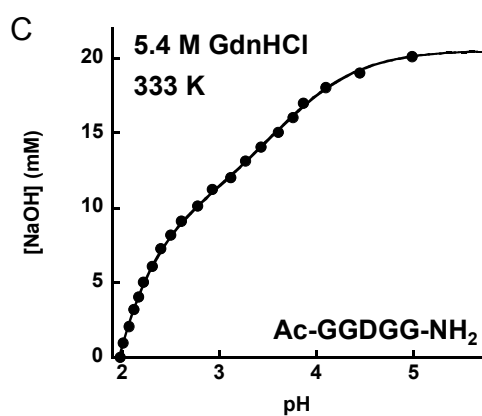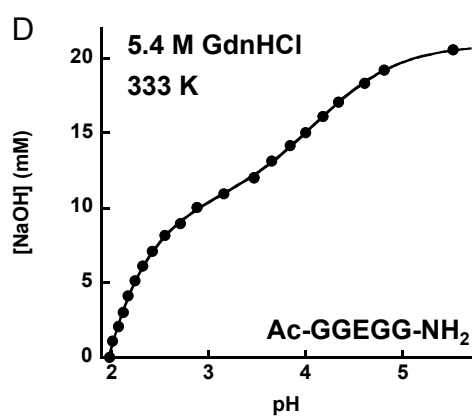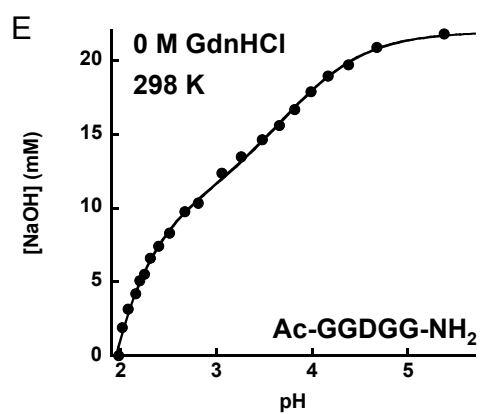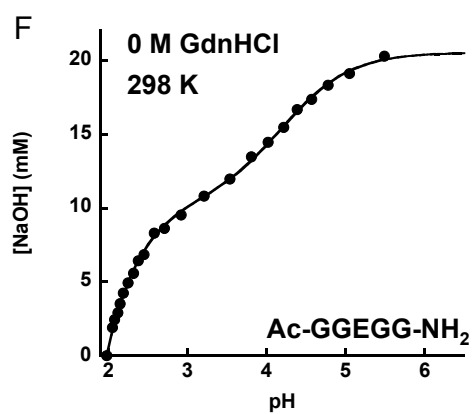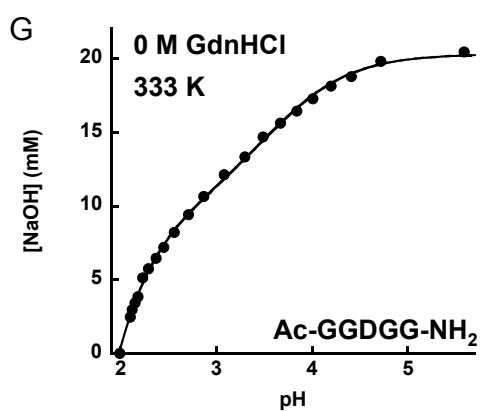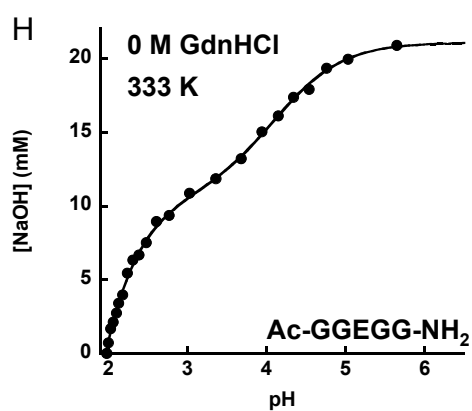

Supplement: Figure S5 — Determination of pKapeptide. Titration curves of model peptides (A, C, E, G) Ac-GGDGG-NH2 and (B, D, F, H) Ac-GGEGG-NH2 in the presence of (A–D) 5.4 M and (E–H) 0 M guanidine HCl (A, B, E, F) 298 K and (C, D, G, H) 333 K. All of the titration data were fitted to the standard Henderson-Hasselbalch equation for determination of the pKa values of Asp and Glu in model peptides. (PDF) [file pone.0030296.s005.pdf]

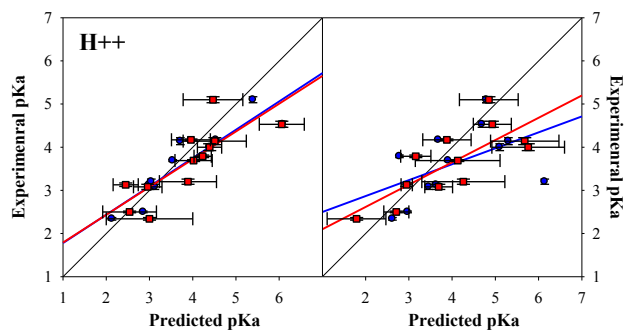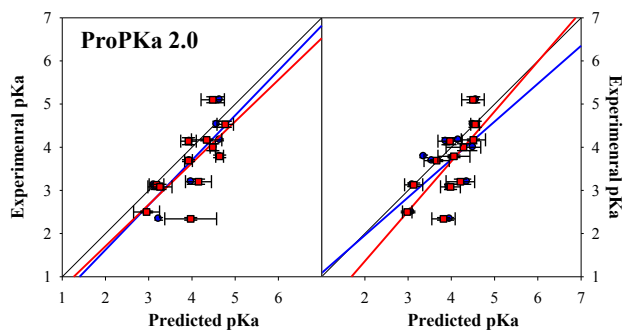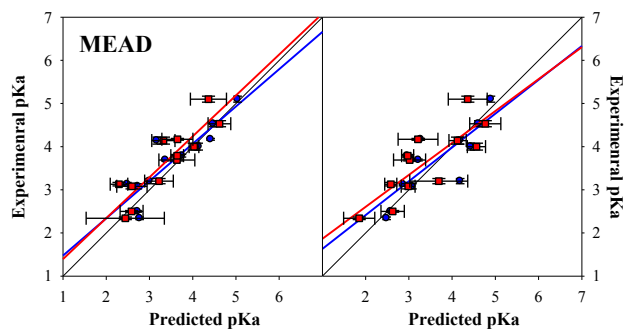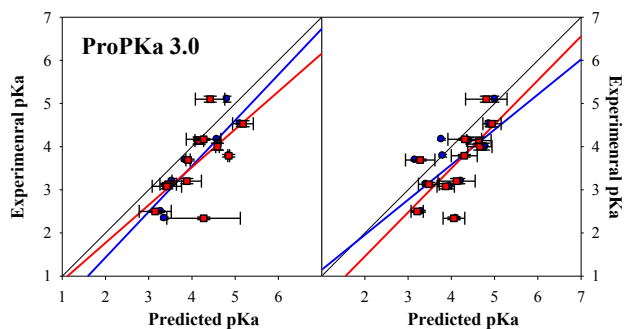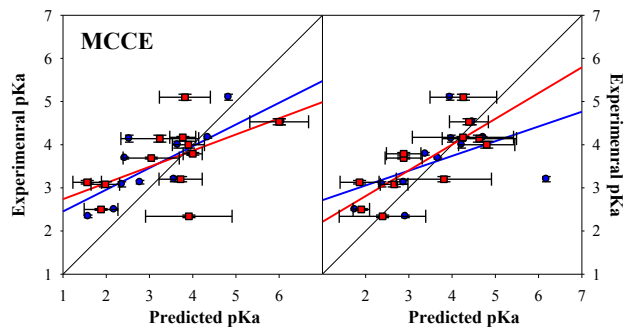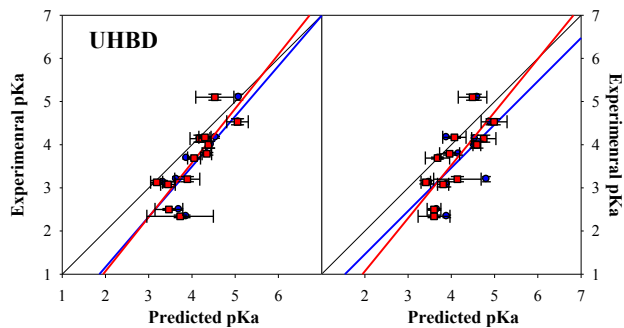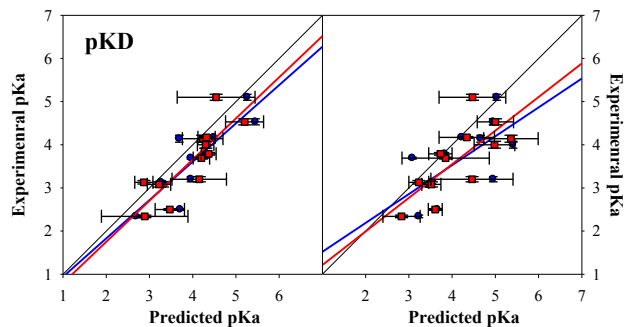

Supplement: Figure S6 — Correlation of experimental and predicted pKa values. pKa values were calculated using several different software packages indicated on each plot. Left panels for each method are based on chain A of the crystal structure of L30e* (PDB code: 3N4Z), right panels for each method are based on chain B. Blue circles represent the results of based on x-ray model (A or B). Red squares represent the results of ensemble calculations based on structures A or B. Red and blue lines are the corresponding linear correlations. Thin black line shows the perfect correlation. See Materials and Methods section for the details of the calculations. (PDF) [file pone.0030296.s006.pdf]
