# Supplementary material for: Electrostatic Contribution of Surface Charge Residues to the Stability of a Thermophilic Protein: Benchmarking Experimental and Predicted pKa Values
Source: PLoS One. 2012 Jan 18;7(1):e30296. doi: 10.1371/journal.pone.0030296 (PMC3261180; doi:10.1371/journal.pone.0030296)
Supplement: Table S1 — Statistics for structure determination of wild-type T. celer L30e and 5R→K variant. (PDF) [file pone.0030296.s007.pdf]

Table S1. Statistics for structure determination of wild-type T. celer L30e and 5R→K variant

	**Wild-type (PDB: 3N4Y)**	**5R→K (PDB: 3N4Z)**
**Summary of crystallization conditions:**		
	10 mM citrate / phosphate, pH 6.5, 298 K	1.6 M Na / K phosphate, pH 7.5, 289 K
**Diffraction data collection statistics:**		
X-ray source	Cu Kα	Cu Kα
Space group	$P2_1$	$P22_12_1$
Resolution (Å)	31.9 – 2.4 (2.5 – 2.4)	39.4 – 2.4 (2.5 – 2.4)
Molecules per asymmetric unit	1	2
Unit cell dimension (Å)	a, 24.2; b, 53.3; c, 33.7	a, 30.5; b, 61.4; c, 102.8
Unit cell angles (deg.)	α, 90.0; β, 109.1; γ, 90.0	α, 90.0; β, 90.0; γ, 90.0
Multiplicity	3.5 (3.5)	14.7 (14.7)
Completeness (%)	99.8 (99.8)	100.0 (100.0)
Mean $I/\sigma(I)$	6.7 (3.9)	15.4 (8.7)
R_{merge} (%)	12.6 (26.4)	12.6 (26.8)
Unique reflections	3199 (456)	8086 (1149)
**Structural refinement statistics:**		
R-factor / R_{free} (%)	18.7 / 27.8	18.9 / 25.0
r.m.s.d. from ideal values:		
Bond distances (Å)	0.006	0.007
Bond angles (deg.)	0.893	1.069
Ramachandran plot analysis		
Preferred region (%)	94.7	98.4
Allowed region (%)	5.3	1.6
Outliers (%)	0.0	0.0
C^{α} r.m.s.d. (1H7M)	0.44	0.47

Values in parentheses are for the highest-resolution shell.
